# Sex differences in auditory brainstem response audiograms from vasopressin-deficient Brattleboro and wild-type Long-Evans rats

**DOI:** 10.1371/journal.pone.0222096

**Published:** 2019-08-30

**Authors:** Payton E. Charlton, Kelcie C. Schatz, Kali Burke, Matthew J. Paul, Micheal L. Dent

**Affiliations:** Department of Psychology, University at Buffalo, The State University of New York, Buffalo, New York, United States of America; Universidad de Chile, CHILE

## Abstract

Rats are highly social creatures that produce ultrasonic vocalizations (USVs) during social interactions. Brattleboro rats, a Long-Evans derived rat that lacks vasopressin (AVP) due to a mutation in the *Avp* gene, exhibit atypical social behavior, including fewer USVs with altered spectrotemporal characteristics during social interactions. It is unclear why Brattleboro rats produce atypical USVs, but one factor could be differences in auditory acuity between them and wild-type Long Evans rats with functional vasopressin. Previous studies have suggested a link between increased levels of AVP and auditory processing. Additionally, few studies have investigated sex differences in auditory perception by Long-Evans rats. Sex differences in auditory acuity have been found throughout the animal kingdom, but have not yet been demonstrated in rat audiograms. This study aimed to measure auditory brainstem response (ABR) derived audiograms for frequencies ranging from 1 to 64 kHz in male and female homozygous Brattleboro (Hom), heterozygous Brattleboro (Het), and wild-type (WT) Long-Evans rats to better understand the role of AVP and sex differences in auditory processing by these rats. We failed to detect significant differences between the ABR audiograms of Hom, Het, and WT Long-Evans rats, suggesting that varying levels of AVP do not affect auditory processing. Interestingly, males and females of all genotypes did differ in their ABR thresholds, with males exhibiting higher thresholds than females. The sex differences in auditory acuity were significant at the lowest and highest frequencies, possibly affecting the perception of USVs. These are the first known sex differences in rat audiograms.

## Introduction

Vocal communication is thought to serve a key role in facilitating or suppressing different behaviors, such as approach behaviors and play. In order for vocal communication to occur, there are two roles that need to be present: a sender (the animal producing the vocalization) and a receiver (the animal perceiving and processing the vocalization). In order for vocal communication to be effective, the sender needs to be able to perceive and decipher the auditory signals being produced. Theories describing the co-evolution of communication signals and sensory systems involve exploiting the environment in which animals live to send conspicuous signals or exploiting the latent preferences of certain signals [1,2]. A key aspect of communication systems is a drive to maximize the signal to noise ratio for the receiver of the signal. This maximization can be accomplished by increasing a signal’s intensity or amplitude, producing vocal signals that degrade at a certain rate based on distance from the receiver, or by producing signals in environments with little ambient noise. Additionally, the signal to noise ratio can also be maximized by biological considerations such as auditory tuning of spectral (frequency) or temporal (duration) features of the signal and the ability to average a signal over time [1]. Regardless of how signals evolve, and whether the evolution is sender driven or receiver driven, the design of signals is not arbitrary and the differences between signals are important [3].

Rats (*Rattus norvegicus domesticus*) are highly social creatures that emit ultrasonic vocalizations (USVs) during social interactions such as rough-and-tumble play behavior [4–8]. These USVs likely serve a communicative function within different social contexts. Rat USVs have been broadly classified into two categories: 22 kHz and 50 kHz [5,6]. The 22 kHz USVs are monotonous vocalizations ranging from 20–30 kHz, and are thought to communicate distress, an aversive state [5,9], or alarm (reviewed in [10]). The 50 kHz USVs are short in duration and are thought to communicate positive, appetitive states, and are considered prosocial [5, 11–13].

In order for USVs to serve a role in social interactions, it is imperative that rats are able to perceive the incoming signals. Wild-type Long-Evans rats have a peak auditory sensitivity between 8 and 16 kHz for pure tones [14,15]. This is comparable to other rat strains such as Fischer 344, Wistar, Sprague-Dawley, and Hooded Norway rats (reviewed in [16]). Auditory thresholds for different frequencies often vary between different strains of rats [16,17]. Additionally, the peak frequency of vocalizations often correlates with peak auditory sensitivity within a species [18–20]. For example, peak auditory sensitivity corresponds to the peak/dominant frequency in songs of Belgian Waterslager canaries (*Serinus canaria* [20]), calls of the orange-fronted conure (*Aratinga canicularis* [21]) vocalizations from four Australian pygopod geckos (*Delma desmosa*, *Delma fraseri*, *Delma haroldi*, *Delma pax* [19]), vocalizations of several anabantoid fishes (*Trichopsis vittata*, *Trichopsis pumila*, *Colisa lalia*, *Macropodus opercularis*, *Trichogaster trichopterus* [22]), vocalizations from Lusitanian toadfish (*Halobatrachus didactylus* [23]) and vocalizations from Chinese webbed-toed geckos (*Gekko subpalmatus* [24]).

There is some evidence that vasopressin (AVP) plays a role in auditory perception. Vasotocin, the non-mammalian homolog of AVP, is found in auditory processing regions of bullfrogs (*Rana catesbeiana* [25]) and plainfin midshipman fish (*Porichthys notatus* [26–28]). Singing mice (*Scotinomys teguina* and *Scotinomys xerampelinus*) have vasopressin 1a (V1a) receptor expression in the medial geniculate nucleus, an area that plays a key role in auditory processing in mammals [29]. In Lincoln’s sparrows (*Melospiza lincolnii*), vasotocin-immunoreactivity is correlated with the quality of songs they hear [30]. AVP could also impact sensitivity in the inner ear, where it binds to V2 receptors to increase water permeability [31–35]. Altered water permeability can lead to excess water in the inner ear, which can negatively impact hearing [31,34,36].

AVP also regulates several social behaviors, including play, social recognition, parental behavior, aggression, and vocal communication in several strains of Wistar, Sprague-Dawley, and Long-Evans laboratory rats, singing mice, Syrian hamsters (*Mesocricetus auratus*), house rats (*Rattus rattus*), prairie voles (*Microtus ochrogaster*), meadow voles (*Microtus pennsylvanicus*), European hamsters (*Cricetus cricetus*), garden dormice (*Eliomys quercinus*), wild house mice (*Mus domesticus*), guinea pigs (*Cavia porcellus*), and the Greater Egyptian jerboa (*Jaculus orientalis*) [37–40]. Central injections of AVP increase maternal separation induced USVs of laboratory rat pups [41]. Conversely, pharmacological or genetic disruptions to AVP decrease maternal separation induced USVs of infant CD and Brattleboro strain rat pups [42–44], 50 kHz USVs of juvenile Brattleboro and Wistar rats emitted during affiliative behaviors, and USVs of adult female vasopressin 1b knockout mice emitted during aggressive interactions [45–47].

The Brattleboro rat is an ideal laboratory model to study the impact of lifelong disruptions to AVP. Brattleboro rats have a mutation in the *Avp* gene and lack AVP throughout life [48]. This mutation has been maintained on the Long Evans background strain of laboratory rat. Consistent with AVP’s role in social behavior, Long Evans rats homozygous for the Brattleboro mutation (Hom) exhibit atypical social behaviors characterized by decreased social interactions (including juvenile social play), decreased 50 kHz USVs, but increased huddling compared to both heterozygous (Het) siblings that have one copy of the Brattleboro mutation and wild-type (WT) siblings that do not carry the mutation (i.e., have two copies of the functional AVP gene) [46,49,50]. Furthermore, the spectrotemporal characteristics of their 50 kHz vocalizations differ from the vocalizations of their Het and WT littermates. Brattleboro rats’ upward-ramp, flat, step-up, and complex USVs had lower integrated frequencies than the other two genotypes, while step-up calls and trills differed in durations across the three genotypes [46]. Adult Brattleboro rats have deficits in their event-related potentials to auditory stimuli [49] suggesting that the Brattleboro mutation may also impact auditory processing and/or perception. Altered acoustic communication in the Brattleboro rats could contribute to their atypical social phenotype.

Males have higher levels of AVP expressing cells in the bed nucleus of the stria terminalis and medial amygdala, as well as denser projections to fore-, mid-, and hindbrain regions than females [51]. The sex difference in this pathway is driven by organizational actions of perinatal androgens, activational actions of adult gonadal steroids, and direct effects of genes on the sex chromosomes [52]. Sex differences in AVP are thought to regulate sex differences in some behaviors, such as social behaviors, and manipulations of AVP can have different, sometimes opposite, actions. For example, septal infusion of a V1a receptor antagonist increases social play in juvenile male rats, but decreases social play in females [53,54]. Similarly, septal AVP infusion enhances social recognition in juvenile male rats, but has no effect in females [55]. For these reasons, it is important to determine whether AVP manipulations (e.g., lack of AVP in Brattleboro rats) have a similar impact in males and females.

Females and males often differ in their sensory processing due to differing genetic material and exposure to gonadal hormones during development [56,57]. Female mammals tend to have more sensitive auditory thresholds than males, and females tend to retain their hearing for longer over their lifespan [58,59]. There are sex differences in the audiograms of many strains of mice, and these sex differences are often compounded by noise exposure and age (reviewed in [60]). The heightened sensitivity and longer retention of auditory acuity in females is believed to be due to the protective actions of estradiol [58]. In humans, menopausal women given hormone therapy demonstrated better auditory acuity relative to control women who had received no hormone therapy [61]. This was similar to findings from ovariectomized rats that received estrogen replacement [62].

In the present experiment, we tested whether there are sex differences in the auditory acuity of Long-Evans laboratory rats. We further asked whether lifelong disruptions in AVP impact auditory sensitivity or potential sex differences in auditory acuity, i.e., whether ABRs of Long Evans rats carrying the Brattleboro mutation would differ from WT Long Evans rats. We reasoned that if AVP plays a critical role in sensitivity, Hom rats would have higher auditory thresholds (lower acuity) than WT rats, or would exhibit a shifted peak sensitivity to frequencies that match the lower frequency of their USVs. To test these hypotheses, auditory brainstem responses (ABRs) were measured in male and female Hom, Het, and WT littermates. ABRs demonstrate how the cochlea and auditory pathways are working when presented with different frequencies at different intensities. The waveforms that are produced allow us to determine both hearing thresholds and peak sensitivity, which can then be compared across sexes and genotypes.

## Materials and methods

### Ethics statement

All procedures were approved by the University at Buffalo, SUNY’s Institutional Animal Care and Use Committee [IACUC] and were in accordance with the *Guide for Care and Use of Laboratory Animals*.

### Subjects

Male and female wild-type Long-Evans rats (WT; n = 10; 5 males, 5 females) and those homozygous (Hom; n = 10; 5 males, 5 females) or heterozygous (Het; n = 10; 5 males, 5 females) for the Brattleboro mutation were obtained from our breeding colony maintained at the University at Buffalo, State University of New York, which were derived from rats obtained from the Rat Resource and Research Center (University of Missouri, Columbia, MO). Breeding pairs consisted of Het males and Het females in order to produce subjects from all three genotypes within the same litters. Offspring were ear punched on postnatal day (P)14, and ear tissue was used for genotyping (see genotyping procedures below). Rats were weaned into same-sex, same-genotype pairs on P21. All rats were housed in plastic cages (44 cm X 22.5 cm X 20.5 cm) with corn cobb bedding (Envigo) and had *ad libitum* access to food and water.

### Genotyping

Genotyping was performed using the method described in Paul et al. [46]. This procedure has been validated by sequencing [46] as well as behavioral phenotyping of water intake [63]. Briefly, ear tissue was digested and DNA extracted using Extraction Solution and Neutralization Solution B from the REDExtract-N-Amp Tissue PCR Kit (Sigma-Millipore). DNA surrounding the Brattleboro mutation was amplified by PCR using GoTaq Green Master Mix (Promega), the forward primer GACGAGCTGGGCTGCTTC, and the reverse primer, CCTCAGTCCCCCACTTAGCC. Samples were subsequently incubated overnight at 37°C with the restriction endonuclease, Bcg1 (New England BioLabs), which cuts the Brattleboro allele, but not the wild-type allele. Samples were run on a 2% agarose gel for visualization of DNA fragments corresponding to the Hom, Het, and WT genotypes. Samples from WT rats exhibit a single 222-bp band, whereas those of Hom rats exhibit a single 95-bp band (the two fragments do not separate on a 2% agarose gel). Samples from Het rats exhibit both WT and Hom bands.

### ABR procedure

Auditory brainstem responses were collected one month apart from each other from two groups of rats. Thirty rats were tested between 50 and 60 days of age. Rats were anesthetized with a mixture of 90 mg/kg ketamine and 10 mg/kg xylazine. All rats were anesthetized at a dose between 60–100% of their body weight in order to keep them fully sedated for the duration of the procedure.

Rats were placed on a heating pad kept at 37°C inside a small sound-attenuated chamber (interior dimensions 55 x 33 x 36 cm) lined with 4 cm thick Sonex sound-attenuating foam (Illbruck Inc., Minneapolis, MN). The ABR system was manufactured by Tucker-Davis Technologies (TDT, Alachua, FL). The test chamber contained the needle electrodes (ELE-N), a speaker (MF1), a 4-channel preamplifier (RA4PA), a 4-channel low impedance headstage (RA4LI), a heating pad, and the test subject. The speaker was placed three inches away from the ear being stimulated. Subcutaneous needle electrodes were placed on the bulla of alternating ears (approximately half on the left bulla, half on the right bulla, randomly assigned), on the vertex of the skull, and a ground electrode was inserted into the opposite leg of the bulla being stimulated.

The experiments were controlled by a WS4 Windows computer running an Optibit interface on a TDT driver using BioSigRZ. The stimuli used in this experiment were generated by a Multi I/O Processor with optic port (RZ6-A-P1) and sent to the speaker. Digitized data from the preamplifier were sent back to the RZ6 processor. The noise floor for each subject was obtained prior to testing by placing the electrodes and recording activity in with no stimulus presentation.

Broadband sounds 0.1 ms in duration (defined as “clicks”) were first tested to verify electrode placement and a clearly observable response. The clicks were presented at a rate of 21 presentations per second. A total of 512 responses were averaged at each sound pressure level. Sounds were calibrated using a quarter-inch free field microphone (PCB-378C01) placed at the location of the rat’s head using TDT software. Clicks were presented at 90 dB SPL, descending in 5 dB steps. Tones were then presented in ascending order from 1 to 64 kHz. Frequencies tested were 1, 2, 4, 8, 16, 24, 32, 42, and 64 kHz. Each tone was presented at a rate of 21 presentations per second. A total of 512 responses were averaged for each frequency at each sound pressure level. The tones were 5 ms in durations, and were cosine-gated tones. Tones were then presented beginning at 90 dB SPL, descending in 10 dB steps. Testing lasted approximately 45 minutes, after which rats were placed on a heating pad until they regained consciousness and then returned to their home cage.

### Data analysis

ABR waveforms were bandpassed between 500 Hz and 3 kHz. Thresholds were quantitatively determined as the average value between the lowest intensity where a waveform response is still qualitatively present and the intensity where no waveform response is qualitatively present.

A three-way mixed analysis of variances (ANOVA) was conducted on the final thresholds, with sex and genotype as between-subject factors and frequency as a within-subjects factor. All possible post hoc tests were conducted using the Bonferroni correction. Significance was assumed when *p* < .05. Statistical analyses were conducted using SPSS software, Version 24. Five males and five females for each of the three genotypes were included in the statistical analyses, resulting in 10 rats for each genotype, for a total of 30 rats. These results were graphically compared to previous results from Popelar et al. [15] to demonstrate similar ABR thresholds across studies. Post-hoc power analyses were conducting using G*Power, version 3.1.9.4.

## Results

**Fig 1:**
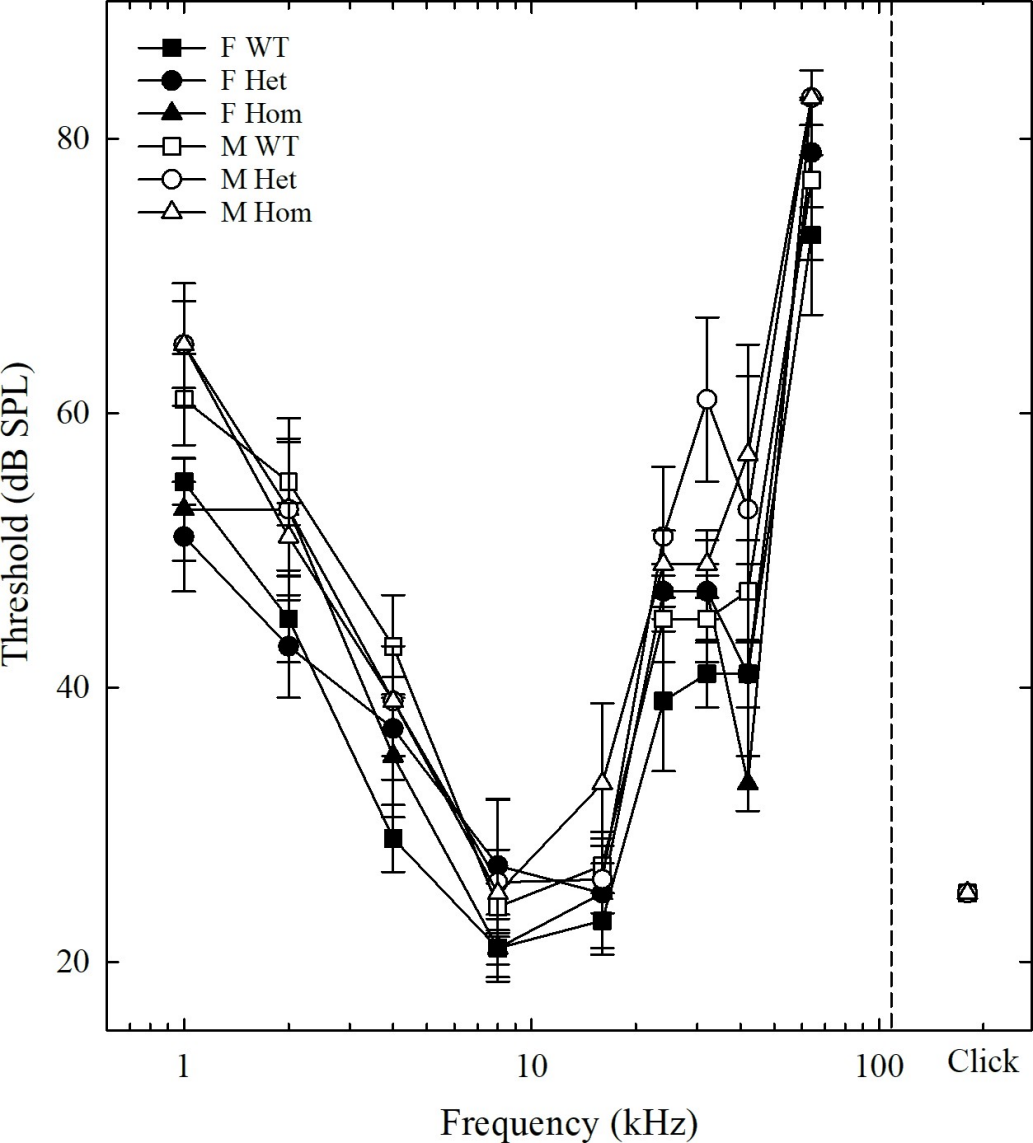
Auditory brainstem thresholds across genotype and sex. Mean ABR thresholds for each genotype and sex for tones ranging from 1 to 64 kHz and clicks. Error bars are standard error of the mean (Males = white fill, Females = black fill, wild-type = squares, heterozygotes = circles, and Brattleboro homozygotes = triangles).

**Fig 2:**
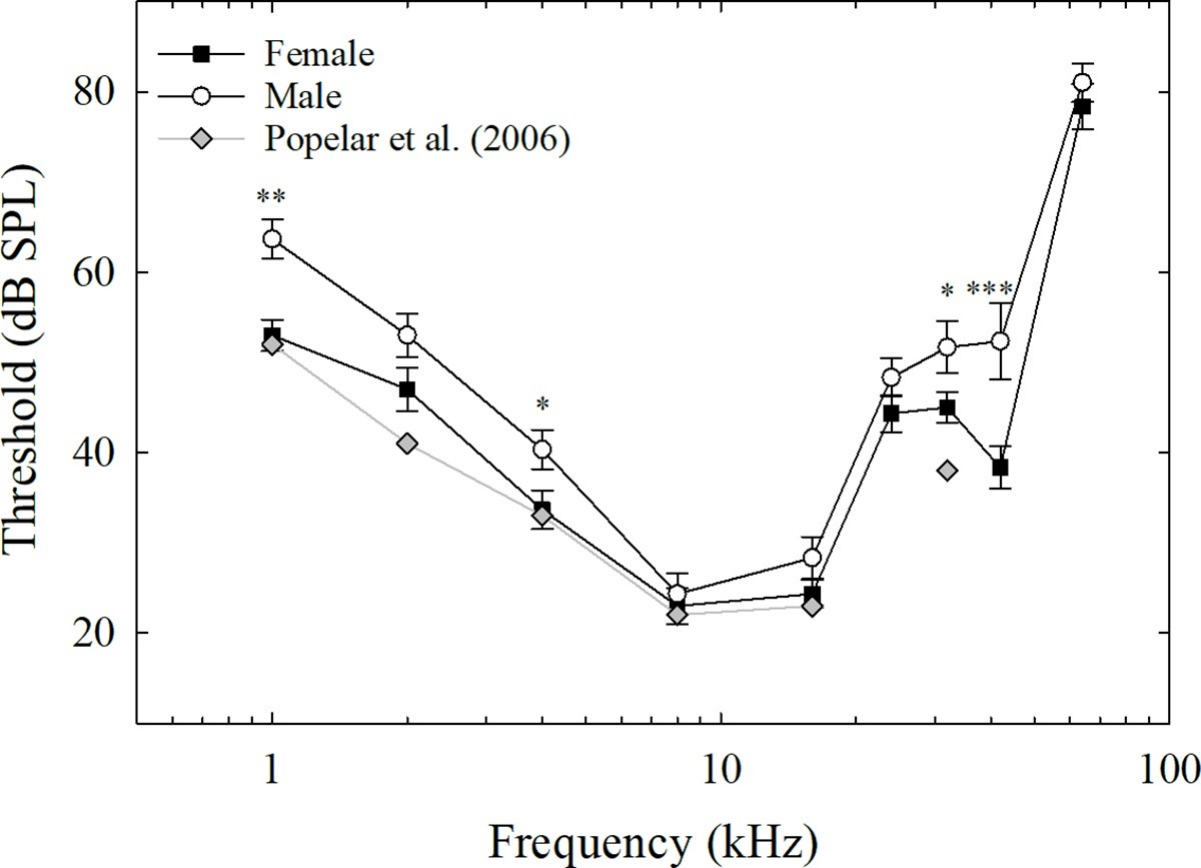
Auditory brainstem thresholds across sex. Mean ABR thresholds for males and females across all three genotypes for tones ranging from 1 to 64 kHz (error bars are standard error of the mean) compared to Popelar et al. [15] ABR thresholds from 1 month old male Long-Evans rats (Females = black squares, Males = white circles, and Popelar et al. (2006) = gray diamonds). *p < .05, **p < .01, ***p < .001.

ABR audiograms were constructed for male and female mice across the three genotypes separately (see Fig 1). Thresholds differed across the six groups by as little as 6 dB at 8 kHz and by as much as 24 dB at 42 kHz. Thresholds for the click stimuli were exactly the same for all six groups: 25 dB. Peak sensitivity for all six groups was between 8 and 16 kHz, with higher thresholds at higher and lower frequencies. Fig 2 illustrates the mean audiograms for males and females collapsed across the three genotypes as well as ABR audiograms from juvenile male wild-type Long-Evans rats (from [15]). The patterns of ABR audiograms across frequencies, including frequencies of peak sensitivity and overall acuity, were similar between the current study and the previous one from Popelar et al. [15].

There were significant main effects of frequency and sex, but not genotype (main effect of frequency: *F*(8, 27) = 151.00, *p* < .001, ηp^2^ = .863; main effect of sex: *F*(1, 27) = 8.81, *p* = .007, ηp^2^ = .269; main effect of genotype: *F*(2, 27) = 1.13, *p* = .340, ηp^2^ = .086). There was a significant interaction between frequency and sex (*F*(8, 27) = 2.11, *p* = .037, ηp^2^ = .081). No significant interaction between frequency and genotype was found (*F*(16, 27) = 1.02, *p* = .442, ηp^2^ = .078). There was no significant three-way interaction between sex, genotype, and frequency (*F*(16, 27) = 1.47, *p* = .113, ηp^2^ = .109) (see Fig 1). Bonferroni post-hoc tests revealed significant mean differences amongst several frequencies. These results are outlined below in Table 1.

**Table 1:** Mean thresholds (M), differences between thresholds (dB SPL, Cell Values), and bonferroni post-hoc test results (* where significant).

Frequency	M (SEM)	1 kHz	2 kHz	4 kHz	8 kHz	16 kHz	24 kHz	32 kHz	42 kHz	64 kHz
1 kHz	58.33 (1.68)									
2 kHz	50.00 (1.78)	8.33***								
4 kHz	37.00 (1.62)	21.33***	13.00***							
8 Hz	23.67 (1.50)	34.67***	26.33***	13.33***						
16 kHz	26.33 (1.42)	32.00***	23.67***	10.67***	-2.67					
24 kHz	46.33 (1.50)	12.00***	3.67	-9.33**	-22.67***	-20.00***				
32 kHz	48.33 (1.75)	10.00***	1.67	-11.33***	-24.67***	-22.00***	-2.00			
42 kHz	45.33 (2.69)	13.00***	4.67	-8.33*	-21.67***	-19.00***	1.00	3.00		
64 kHz	79.67 (1.64)	-21.33***	-29.67***	-42.67***	-56.00***	-53.33***	-33.33***	-31.33***	-34.333***	

* *p* < .05

** *p* < .01

*** *p* < .001

Bonferroni post-hoc tests were conducted to probe the significant interaction between sex and frequency. These tests revealed that males and females significantly differed in threshold values for 1 kHz (*p* = .002), 4 kHz (*p* = .046), 32 kHz (*p* = .046), and 42 kHz (*p* < .001). Females and males did not differ in threshold values for 2, 8, 16, 24, and 64 kHz (*p* > .05) (see Fig 2).

A post-hoc power analysis using a repeated-measures within-subjects design was conducted on the main effect of frequency and yielded a power of 1.00. Post-hoc power analyses using a repeated-measures between-subjects design were conducted on the main effects of sex and genotype and yielded a power of 0.99 and 0.46, respectively. Post-hoc power analyses using a repeated-measures within-between interaction design were conducted on the interactions between frequency and sex, frequency and genotype, and frequency, sex, and genotype. Both the interaction between sex and frequency and the interaction between genotype and frequency yielded a power of 0.99. The three-way interaction between frequency, sex, and genotype yielded a power of 0.96.

## Discussion

The goals of this study were to determine whether there are sex differences in the auditory acuity of Long-Evans rats and whether the ABR thresholds are altered by the Brattleboro mutation. There was a significant main effect of sex on pure tone thresholds, in addition to a significant interaction between sex and frequency. Males generally had higher thresholds at the low and high ends of the frequencies tested compared to females, specifically for 1, 4, 32, and 42 kHz. Sex differences in auditory acuity and auditory anatomy are common in the animal kingdom, found in humans, mice, and birds, to name a few [59, 64–66]. However, to our knowledge, this is the first demonstration of sex differences in ABR-derived thresholds of laboratory rats. Sex differences in auditory acuity are typically amplified as animals age, possibly due to the protective effects of estrogen on the auditory system. It is unknown if the sex differences found here will increase in older rats, or if there would be an interaction between the Brattleboro rats and their wild-type Long Evans counterparts with age.

Significant sex differences in the ABRs were not found at all frequencies, but were obtained at some of the lowest and some of the highest frequencies tested. It is not known whether the sex differences occurred at ecologically “meaningful” frequencies. Neither 1 kHz nor 4 kHz are typically employed in rodent vocalizations, although 4 kHz falls within the range of audible rodent “squeals.” These “squeals” are usually produced in times of distress and are thought to express physical pain or discomfort [67]. The sex differences in the 32 and 42 kHz ABR thresholds are interesting, as these are typical frequencies found in rat USVs. However, rats in this study were tested with pure tones rather than USVs, so while the pure tone frequencies align with USV frequencies, we cannot say definitively whether there are differences in USV detection between male and female Long-Evans rats using the results from the current study.

The sex differences in auditory acuity are possibly linked to the sex differences in vocal production in rats. Previous studies have shown that male rat pups produce a greater quantity of USVs than females do, and their USVs tend to be slightly lower in fundamental frequency and amplitude than USVs from females [68]. Males also produce more USVs during play bouts than females do [69], although another report only found this to be true for trills [46]. Additionally, Graham and colleagues [70] found sex differences in USVs in two different strains of rats, with males producing more USVs than females (Sprague-Dawley and Long-Evans).

Sex differences in vocalizations are not limited to rats. Male golden hamsters (*Mesocricetus auratus*) produce shorter USVs than females, and their USVs had lower entropy and bandwidth compared to female USVs [71]. Mice are also known to produce variable vocalizations across the sexes, differing in spectrotemporal characteristics [72,73], amplitude [74], and proportions of call categories produced [72]. Responses to USVs from playback studies also commonly show sex differences (e.g., [75]). Although the function of mouse USVs is not entirely known, there are clear parallels across rodent species. As USVs are typically produced alongside social behaviors in rats, the sex differences observed in USV production and social behaviors align with each other.

Another possibility for the presence of sex differences in auditory acuity could be that the young female Long-Evans rats’ ABR thresholds are influenced by levels of estradiol. Estradiol has been linked to auditory perception and processing changes in young and old female laboratory rats, mice, and humans (as reviewed in [76]). Older human females (post-menopausal) have higher ABR thresholds than young females and young males [77,78]. Additionally, ovariectomized laboratory rats also show higher ABR thresholds compared to non-ovariectomized rats [62]. Estradiol has also been found to affect outer hair cells, as young females have larger otoacoustic emission (OAEs) amplitudes than young males [79,80]. Furthermore, females who use oral contraceptives have been found to produce fewer spontaneous OAEs (SOAEs) and lower click-evoked OAE (CEOAE) amplitudes than females who did not use oral contraceptives [81,82]. These findings are likely due to differing levels of estradiol and other steroid hormones, such as testosterone. It is possible that the sex differences in auditory acuity exhibited by the Long-Evans strain of rat could be due these hormone effects. Studies manipulating gonadal steroid hormones and measuring SOAEs, CEOAEs, and distortion product OAEs (DPOAEs) alongside ABRs would be a fruitful avenue for research into sex differences in hearing acuity.

Since AVP has been linked to auditory processing [37], we hypothesized that auditory thresholds, as measured by ABR audiograms, would differ amongst Hom, Het, and WT Long-Evans rats. When Naganuma and colleagues [83] injected AVP and then measured ABRs, thresholds were significantly elevated. Counter to our hypothesis, however, we failed to detect a significant difference across the three genotypes, suggesting that the chronic vasopressin deficiency does not influence auditory acuity. Our results may have differed from Naganuma and colleagues’ results as they tested acute increases in AVP rather than chronic AVP deficiency. Differences could also arise due to the differing natures of the manipulations–peripheral AVP manipulation from an IP injection versus global AVP loss due to a genetic mutation. In addition, the Brattleboro mutation could trigger compensatory mechanisms during development that ameliorate deficits in auditory detection. Finally, it is possible that a larger sample sizes is necessary to detect genotype differences. Our post-hoc power analysis conducted on the main effect of genotype revealed that our sample size of 30 rats (10 rats per genotype) was moderately powered (β = 0.46).

One possible limitation of the current study is related to the age range in which the rats were tested. Paul and colleagues [46] found differences in the USVs of Brattleboro and wild-type rats at P34 and P44, which corresponds to early and mid-adolescence. However, the ABR audiograms measured in the current study were collected in late adolescent rats, between P50 and P60. Given that adolescence is a time of marked behavioral and neural change, it is possible that between these two time points, differences in USVs became diminished and, in conjunction, differences in auditory acuity could have diminished or disappeared. However, the range at which ABRs were collected still fell within late adolescence and, while USVs could develop quite rapidly, it is unknown whether auditory thresholds would significantly change after 16–20 days after birth [84].

Another possible limitation of the study is that only a few frequencies were tested in the ultrasonic range (24, 32, 42, and 64 kHz). Additionally, these results reflect auditory processing of pure tones, which are not fundamentally the same as USVs. While this study reveals that the basic auditory processing of pure tones and clicks by anesthetized Hom, Het, and WT Long-Evans rats is equivalent, future studies should measure the perception of more tones in the ultrasonic range, as well as USVs, across more ages. Researchers should also measure ABR audiograms in these rats between P34 and P44 when the differences in USVs are most prominent, as well as at other developmental periods to see if the developmental rate changes.

The above findings do not support the idea that differences in auditory perception contribute to differences in USV production or social behaviors, but further studies are needed before this idea can be completely ruled out; Brattleboro rats may show deficits in acuity of natural stimuli. Furthermore, researchers should also investigate other ways of manipulating AVP, such as vasopressin receptor blockers, to determine their effects on acoustic communication. For instance, AVT can influence acoustic communication in amphibians by changing acoustic features in calls or altering the rate of calls (reviewed in [85, 86]). Male túngara frogs (*Physalaemus pustulosus*) treated with exogenous AVT alter the “whine” component of their advertisement calls by increasing the initial frequency and shortening the duration of the call [87]. The altered “whine” call decreases the likelihood of a female túngara frog finding the “whine” attractive and diminishing their preference for the caller [87]. However, exogenous AVT injections also increased the number of “chucks” in male túngara advertisement calls, which can increase attractiveness of male túngara frogs to female túngara frogs [88]. Additionally, exogenous AVT injections in the gray treefrog (*Hyla versicolor*) altered advertisement calls by increasing the duration and including more pulses to the calls [89]. However, these changes were dependent on close proximity to conspecific males [89]. Tito and colleagues [90] found that exogenous AVT injections increased the likelihood of advertisement calling in the gray treefrog; however, call rate (the number of calls produced within a given time frame) of advertisement calls decreased to half of the baseline call rate and the dominant frequency of the calls increased compared to calls produced by non-AVT-treated males [90]. It is possible that manipulations of AVP in mammals could produce similar changes in mammalian acoustic communication as AVT produces in amphibians.

## Conclusions

This was the first attempt to determine correlates between a chronic lack of AVP and auditory processing. We failed to detect a significant effect of genotype, which suggests that chronic AVP deficiency does not affect auditory acuity, at least to simple tone detection. To our knowledge, this is the first study to report sex differences in auditory thresholds in Long-Evans rats. Furthermore, a greater range of frequencies was tested in the present study, including those in the ultrasonic range, providing a more comprehensive view of auditory thresholds.

## Supporting information

S1 File The raw experimental results for individuals across all conditions. (XLSX)
